# The native ABC-F proteins of *Staphylococcus aureus* do not contribute to intrinsic resistance against ribosome-targeting antibacterial drugs

**DOI:** 10.1093/jac/dkad238

**Published:** 2023-08-16

**Authors:** Luiza H Galarion, James Trigwell, Merianne Mohamad, Jose A Nakamoto, Justin E Clarke, Gemma C Atkinson, Alex J O’Neill

**Affiliations:** Faculty of Biological Sciences, School of Molecular and Cellular Biology, University of Leeds, Leeds LS2 9JT, UK; Faculty of Biological Sciences, School of Molecular and Cellular Biology, University of Leeds, Leeds LS2 9JT, UK; Faculty of Biological Sciences, School of Molecular and Cellular Biology, University of Leeds, Leeds LS2 9JT, UK; Department of Experimental Medical Science, Lund University, Lund, Sweden; Faculty of Biological Sciences, School of Molecular and Cellular Biology, University of Leeds, Leeds LS2 9JT, UK; Department of Experimental Medical Science, Lund University, Lund, Sweden; Faculty of Biological Sciences, School of Molecular and Cellular Biology, University of Leeds, Leeds LS2 9JT, UK

Antibiotic resistance ATP-binding cassette F (ARE ABC-F) proteins are a major cause of acquired resistance to antibacterial drugs that target protein synthesis.^1^ These proteins bind to the ribosome to drive antibiotic release, a mechanism known as target protection,^2^ and thereby mediate resistance to diverse drug classes that act on the 50S subunit (lincosamides, macrolides, oxazolidinones, phenicols, pleuromutilins and streptogramins).^1^

In addition to their role in acquired resistance, it is increasingly apparent that ARE ABC-Fs are an important and common source of *intrinsic* resistance to ribosome-targeting antibiotics in many bacterial species, including pathogens. It has long been known that the intrinsic lincosamide/streptogramin resistance of *Enterococcus faecalis* and *Bacillus subtilis* is attributable to native ABC-F proteins [Lsa(A)^3^ and VmlR,^4^ respectively], and recent years have seen a dramatic accumulation of additional examples of ABC-F-mediated intrinsic resistance that include the Sal proteins in non-aureus staphylococci,^5^ VgaL (Lmo0919) in *Listeria monocytogenes,*^6^ MAB_2355c in *Mycobacterium abscessus*^7^ and CplR in Clostridia.^8^ In addition to providing an explanation for the differing levels of inherent susceptibility to ribosome-targeting antibiotics observed across common bacterial species, understanding such intrinsic ARE ABC-Fs may inform improved approaches to deployment or discovery of antibacterial drugs.

Here, we examined whether the native ABC-F proteins of *Staphylococcus aureus* contribute to the intrinsic background level of resistance to ribosome-targeting antibiotics. This pathogen is the prime exponent of acquired antibiotic resistance mediated by ARE ABC-F proteins,^1^ and as indicated above, other members of the same genus are known to harbour native ARE ABC-Fs^5^; consequently, it seemed entirely possible that native ABC-Fs participate in intrinsic antibiotic resistance in *S. aureus*.

To define the complement of native ABC-F proteins in *S. aureus*, we searched the predicted proteome (GCA_002085525.1) of MRSA strain JE2 downloaded from the NCBI genome database with ABC-F subfamily-specific hidden Markov models^9^ using HMMER v.3.3.2 hmmscan^10^ and an e-value threshold of 1e^−70^. This analysis returned three ABC-F proteins: Uup [ARG45262.1] (previously referred to as EttA^11^), YbiT [ARG45891.1], and YdiF [ARG46606.1]. Whether these proteins provide any degree of intrinsic resistance to ribosome-targeting antibacterial drug classes is unknown; whilst an earlier report assessed the antibiotic susceptibility of a strain in which ARG45262.1 was putatively inactivated,^11^ that study did not test the majority of drug classes that fall within the typical spectrum of resistance for ARE ABC-F proteins. We therefore sought to examine susceptibility to relevant drug classes of strains in which these ABC-F genes had been independently inactivated by transposon (Tn) insertion mutagenesis. The corresponding Tn mutants (NE770, NE293 and NE790, respectively) were sourced from the Nebraska Transposon Mutant Library (https://www.unmc.edu/pathology/csr/research/library.html), and modified by allelic exchange to replace the selectable marker on the Tn (*ermB*) with the kanamycin resistance determinant, *aphA-3*; the rationale for this was that *ermB* itself confers resistance to drug classes that we intended to test. Susceptibility testing by CLSI broth microdilution found no differences between the Tn-inactivation strains and the JE2 parent for lincosamides (clindamycin), macrolides (erythromycin), oxazolidinones (linezolid), phenicols (chloramphenicol), pleuromutilins (tiamulin) and streptogramins (virginiamycin M1/S).

To corroborate this result and exclude the possibility that Tn insertions had not completely inactivated gene function, we generated independent, markerless deletions of the three ABC-F genes in JE2 by allelic replacement using plasmid pIMAYZ.^12^ Again, no difference in antibiotic susceptibility was seen for these strains, even when using concentration increments substantially smaller than those ordinarily employed in susceptibility testing. Failure to detect a change in susceptibility in individual ABC-F deletion mutants could potentially reflect functional redundancy between the encoded proteins; consequently, we employed the same pIMAYZ constructs to sequentially delete all three ABC-F genes in a single strain of JE2. The resultant strain also showed no change in antibiotic susceptibility.

Having established that deletion of native ABC-F genes of *S. aureus*—alone or in combination—has no apparent effect on susceptibility to ribosome-targeting drugs, we took an orthogonal approach to examine whether the encoded proteins could potentially contribute to antibiotic resistance by assessing whether they affect susceptibility under conditions of increased expression. Since the expression of ARE ABC-F genes is often under the control of antibiotic-responsive regulatory elements,^4,8^ we first examined whether challenging *S. aureus* JE2 with a subinhibitory concentration (1/4 MIC) of a ribosome-targeting antibiotic—with a view to inducing ABC-F expression—would serve to reduce susceptibility to that same agent in a subsequent MIC determination. No change in susceptibility to any of the antibiotics was observed under these conditions. We subsequently generated independent artificial overexpression constructs for each of the three ABC-F genes using the strong, tetracycline-inducible expression system on plasmid pRMC2.^13^ Under conditions of maximal induction, no change in antibiotic susceptibility was seen in any case.

Thus, we conclude that in contrast to the situation seen for other medically important Gram-positive bacteria—including other members of the same genus—the native ABC-F proteins of *S. aureus* do not contribute to intrinsic resistance to ribosome-targeting antibacterial drugs.
